# High-quality genome assembly and annotation of live animal vaccine bacteria strains in South Korea

**DOI:** 10.1186/s12863-025-01357-8

**Published:** 2025-09-02

**Authors:** Yeonkyeong Lee, Jin-Ju Nah, Hyun-Ok Ku, Il Jang

**Affiliations:** Veterinary Drugs and Biologics Division, Animal and Plant Quarantine Agency, 177, Hyeoksin 8-ro, Gimcheon-si, Gyeongsangbuk-do Korea

**Keywords:** Hybrid assembly, Live attenuated vaccine, Seed lot system, Erysipelothrix rhusiopathiae, Clostridium chauvoei, Bordetella bronchiseptica, Mycoplasmoides gallisepticum

## Abstract

**Objectives:**

Selecting and managing suitable seed strains according to standard principles is a critical factor in manufacturing high-quality biological products. In this study, we determined the complete genomes of live animal vaccine bacterial strains used in South Korea and comprehensively analyzed their gene annotations. This detailed genomic analysis provides a robust foundation for identifying and verifying whether manufacturers utilize the same seed strains at the strain level. Furthermore, these data serve as a valuable reference to ensure consistent mass production and reliable quality control of biological products.

**Data description:**

Complete genome sequencing and annotation were performed on five bacterial strains (*Erysipelothrix rhusiopathiae* NL-11, *Clostridium chauvoei* B, *Bordetella bronchiseptica* 12-147-AFQ2, *Bordetella bronchiseptica* S-55, and *Mycoplasmoides gallisepticum* str. F) obtained directly from live attenuated vaccines. A hybrid genome assembly approach combining Illumina short reads (NovaSeq 6000) and Oxford Nanopore long reads (MinION Mk1B) was used to obtain complete, high-quality, gap-free, and well-annotated genome sequences. The genome sizes of *E. rhusiopathiae* NL-11, *C. chauvoei* B, *B. bronchiseptica* 12-147-AFQ2, *B. bronchiseptica* S-55, and *M. gallisepticum* str. F were 1.78, 2.40, 5.34, 5.34, and 0.95 Mb, respectively.

## Objectives

One of the most critical principles in the manufacture of high-quality biological products is the selection and systematic management of appropriate seed strains. Following the identification of a suitable strain, the strain is cultivated at a large scale from a single viable unit to create an initial seed lot, from which working seeds are derived to produce vaccine batches. The seed lot system ensures that each subsequent product batch originates from the same master seed lot at a specified passage level, thereby preventing variations caused by repeated subculturing [1, 2]. As a part of the seed lot management system, identity testing is performed to confirm that the strains used in the biological products match the originally registered seed strains.

Among the various methods employed to verify the identity of vaccine strains, whole-genome analysis has been widely adopted in recent years [3–5]. In this study, we performed whole-genome analysis of live bacterial vaccines approved for veterinary use to establish reference genomic data that can support the future identification and quality control of vaccine strains.

### Data description

Five live bacterial vaccine strains were used for whole-genome sequencing: *Erysipelothrix rhusiopathiae* NL-11, *Clostridium chauvoei* B, *Bordetella bronchiseptica* 12-147-AFQ2, *Bordetella bronchiseptica* S-55, and *Mycoplasmoides gallisepticum* str. F. All strains were obtained from licensed live vaccines currently marketed for veterinary use. Each freeze-dried vaccine pellet was dissolved in 1 mL of incubation buffer and subjected to nucleic acid extraction using a Maelstrom 4810 system (TANBead, Taoyuan City, Taiwan) following the manufacturer’s instructions.

To produce a high-quality and complete genome assembly, whole-genome sequencing was performed using a hybrid assembly strategy that integrates shotgun short- and long-read sequencing. Libraries for short-read sequencing were prepared using a NEXTflex^®^ Rapid-DNA Seq Kit V2 with NEXTflex^®^ Unique Dual Index Barcodes (PerkinElmer, Waltham, MA, USA), and sequenced as 150-bp paired-end reads on an Illumina NovaSeq 6000 platform (Illumina, San Diego, CA, USA). For long-read sequencing, libraries were constructed using an SQK-NBD 114.24 Native Barcoding Kit 24 V14 (Oxford Nanopore Technologies, Oxford, UK) and NEBNext^®^ FFPE DNA Repair Mix (New England Biolabs, Ipswich, MA, USA) according to the manufacturer’s instructions. Sequencing was performed on an ONT MinION platform using a FLO-MIN114 flow cell (R10.4.1; Oxford Nanopore Technologies) for > 72 h. FASTQ files were base-called using Guppy software (version 6.5.7) in high-accuracy mode.

The raw sequencing data statistics are provided in Data File 1 (Table 1). For subsequent analyses, the raw Illumina reads were processed using Trimmomatic (version 0.39) [6] to remove low-quality bases and adapters. Nanopore reads with Phred scores of < 7 were filtered using Nanofilt (version 2.8.0) [7]. Trimmed nanopore reads were assembled using Flye (version 2.9.5-b1801) [8]. Subsequently, using the --existing_long_read_assembly option, the Flye outputs were hybrid-assembled using Unicycler (version 0.5.1) [9] along with the trimmed Illumina reads. Plasmids were hybrid-assembled using Plassembler (version 1.0.0) [10], however, the results were excluded because of the absence of *repA* genes in all contigs.

**Table 1:** Overview of data files/data sets

Label	Name of data file/data set	File types (file extension)	Data repository and identifier (DOI or accession number)
Data file 1	Raw data (Illumina and MinION) details	MS Excel file (.xlsx)	Figshare: 10.6084/m9.figshare.28693232 [16]
Data file 2	Statistics of denovo assembly	MS Excel file (.xlsx)	Figshare: 10.6084/m9.figshare.28693232 [16]
Data file 3	Features of specific chromosomes	MS Excel file (.xlsx)	Figshare: 10.6084/m9.figshare.28693232 [16]
Data file 4	Circular map of *Erysipelothrix rhusiopathiae* NL-11	PDF (.pdf)	Figshare: 10.6084/m9.figshare.28693232 [16]
Data file 5	Circular map of *Clostridium chauvoei* B	PDF (.pdf)	Figshare: 10.6084/m9.figshare.28693232 [16]
Data file 6	Circular map of *Bordetella bronchiseptica* 12-147-AFQ2	PDF (.pdf)	Figshare: 10.6084/m9.figshare.28693232 [16]
Data file 7	Circular map of *Bordetella bronchiseptica* S-55	PDF (.pdf)	Figshare: (10.6084/m9.figshare.28693232) [16]
Data file 8	Circular map of *Mycoplasmoides gallisepticum* str. F	PDF (.pdf)	Figshare 10.6084/m9.figshare.28693232 [16]
Data set 1	Illumina raw reads of *Erysipelothrix rhusiopathiae* NL-11	Raw sequence reads (.fastq)	NCBI data: https://identifiers.org/ncbi/insdc.sra:SRR31748505 [17]
Data set 2	MinION raw reads of *Erysipelothrix rhusiopathiae* NL-11	Raw sequence reads (.fastq)	NCBI data: https://identifiers.org/ncbi/insdc.sra:SRR31748506 [18]
Data set 3	Genome sequence of *Erysipelothrix rhusiopathiae* NL-11	Standard fasta files (.fasta)	NCBI data: https://www.ncbi.nlm.nih.gov/nuccore/CP176524/ [19]
Data set 4	Illumina raw reads of *Clostridium chauvoei* B	Raw sequence reads (.fastq)	NCBI data: https://identifiers.org/ncbi/insdc.sra:SRR31748507 [20]
Data set 5	MinION raw reads of *Clostridium chauvoei* B	Raw sequence reads (.fastq)	NCBI data: https://identifiers.org/ncbi/insdc.sra:SRR31748508 [21]
Data set 6	Genome sequence of *Clostridium chauvoei* B	Standard fasta files (.fasta)	NCBI data: https://www.ncbi.nlm.nih.gov/nuccore/CP173238/ [22]
Data set 7	Illumina raw reads of *Bordetella bronchiseptica* 12-147-AFQ2	Raw sequence reads (.fastq)	NCBI data: https://identifiers.org/ncbi/insdc.sra:SRR31748513 [23]
Data set 8	MinION raw reads of *Bordetella bronchiseptica* 12-147-AFQ2	Raw sequence reads (.fastq)	NCBI data: https://identifiers.org/ncbi/insdc.sra:SRR31748514 [24]
Data set 9	Genome sequence of *Bordetella bronchiseptica* 12-147-AFQ2	Standard fasta files (.fasta)	NCBI data: https://www.ncbi.nlm.nih.gov/nuccore/CP173073/ [25]
Data set 10	Illumina raw reads of *Bordetella bronchiseptica* S-55	Raw sequence reads (.fastq)	NCBI data: https://identifiers.org/ncbi/insdc.sra:SRR31748509 [26]
Data set 11	MinION raw reads of *Bordetella bronchiseptica* S-55	Raw sequence reads (.fastq)	NCBI data: https://identifiers.org/ncbi/insdc.sra:SRR31748510 [27]
Data set 12	Genome sequence of *Bordetella bronchiseptica* S-55	Standard fasta files (.fasta)	NCBI data: https://www.ncbi.nlm.nih.gov/nuccore/CP173071/ [28]
Data set 13	Illumina raw reads of *Mycoplasmoides gallisepticum* str. F	Raw sequence reads (.fastq)	NCBI data: https://identifiers.org/ncbi/insdc.sra:SRR31748511 [29]
Data set 14	MinION raw reads of *Mycoplasmoides gallisepticum* str. F	Raw sequence reads (.fastq)	NCBI data: https://identifiers.org/ncbi/insdc.sra:SRR31748512 [30]
Data set 15	Genome sequence of *Mycoplasmoides gallisepticum* str. F	Standard fasta files (.fasta)	NCBI data: https://www.ncbi.nlm.nih.gov/nuccore/CP173072/ [31]

The assembled genomes were evaluated using QUAST (version 5.2.0) [11], and gene predictions and annotations were performed using the Prokka and NCBI-PGAP tools [12, 13]. CGView [14] was used to visualize the complete genome sequences. CheckM (version 1.2.3) [15] was used to assess genome completeness and contamination levels. All tools were used with default parameters, unless otherwise specified.

Our hybrid assembly approach produced a complete, high-quality, single-circular genome for all five bacterial vaccine strains. The de novo assembly statistics and genomic characteristics for each vaccine strain are presented in Data files 2 and 3 (Table 1), respectively.

The genome of *E. rhusiopathiae* NL-11 (CP176524, 1,780,802 bp, 36.45% GC) showed 96.64% completeness, 0% contamination, and contained 1,677 protein-coding genes, 55 transfer RNAs (tRNAs), 12 ribosomal RNAs (rRNAs), and 4 non-coding RNAs (ncRNAs) (Table 1, Data file 4, Data sets 1 and 2).

The genome of *C. chauvoei* B (CP173238, 2,398,298 bp, 28.62% GC) showed 99.38% completeness, 0% contamination, and contained 2,291 protein-coding genes, 68 tRNAs, 21 rRNAs, and 5 ncRNAs (Table 1, Data file 5, Data sets 3 and 4).

The genome of *B. bronchiseptica* 12-147-AFQ2 (CP173073, 5,340,645 bp, 68.08% GC) showed 99.74% completeness, 0% contamination, and contained 5,003 protein-coding genes, 56 tRNAs, 9 rRNAs, and 6 ncRNAs (Table 1, Data file 6, Data sets 5 and 6).

The genome of *B. bronchiseptica* S-55 (CP173071, 5,340,639 bp, 68.08% GC) showed 99.74% completeness, 0% contamination, and contained 5,003 protein-coding genes, 56 tRNAs, 9 rRNAs, and 6 ncRNAs (Table 1, Data file 7, Data sets 7 and 8).

The genome of *M. gallisepticum* str. F (CP173072, 949,575 bp, 31.39% GC) showed 99.08% completeness, 0% contamination, and contained 722 protein-coding genes, 32 tRNAs, 6 rRNAs, and 3 ncRNAs (Table 1, Data file 8, Data set 9 and 10).

### Limitations

Hybrid assembly of the plasmids was performed using Plassembler (version 1.0.0) [10]. However, we were unable to identify a contig containing *repA*. This limitation did not strongly impact the study because the primary objective of this data note is to serve as a reference for the effective management of seed lot systems across the five strains.

Interestingly, based on the NCBI GenBank database, although the reference genomes of four of the five species consisted of only a single chromosome, the reference genome of *C. chauvoei* contains a plasmid sequence. This finding suggests that the *C. chauvoei* B strain analyzed in this study also possesses a plasmid. Further research is required to obtain plasmid-related information, particularly for studies where data on plasmids, such as those linked to antibiotic resistance, are crucial.

## Data Availability

The raw sequencing data generated and analyzed in the current study have been submitted to the NCBI Sequence Read Archive under BioProject accession number PRJNA1178821. The BioSample numbers for each strain are SAMN45851428, SAMN44498921, SAMN44498925, SAMN44498923, and SAMN44498924. The assembled genomes have been deposited in the NCBI GenBank database (accession numbers: CP176524, CP173238, CP173073, CP173071, and CP173072). Please refer to Table 1 for detailed information and direct links to individual datasets.
